# Comparison of muscle activation and kinematics during free-weight back squats with different loads

**DOI:** 10.1371/journal.pone.0217044

**Published:** 2019-05-16

**Authors:** Roland van den Tillaar, Vidar Andersen, Atle Hole Saeterbakken

**Affiliations:** 1 Nord University, Department of Sport Science and Physical Education, Levanger, Norway; 2 Western Norway University, Faculty of Teacher Education, Culture and Sport, Department of Sport, Food and Natural Sciences, Sogndal, Sogn og Fjordane, Norway; University of Belgrade, SERBIA

## Abstract

Although several studies have examined the effects of performing resistance training with different percentages of one-repetition maximum (1-RM), little is known of the neuromuscular effects and kinematics of lifting low to heavy loads with maximal movement velocity. The aim of this study is to compare muscle activation and kinematics in free-weight back squats with different loads. Thirteen resistance-training males (aged 24.2 ± 2.0 years, body mass 81.5 ± 9.1 kg, height 1.78 ± 0.06 m) with 6 ± 3 years of resistance-training experience conducted squats with 30%–100% of 1-RM. Barbell kinematics and electromyographic (EMG) activity of the vastus lateralis, vastus medialis, rectus femoris, semitendinosus, biceps femoris, and gluteus maximus were measured in the upward phase of each load. With increasing loads, the barbell velocity decreased, the upward phase duration increased, and the peak velocity occurred later. The muscle activation in all muscles increased with increasing loads but was not linear. In general, similar muscle activation in the prime movers was observed for loads between 40% and 60% of 1-RM and between 70% and 90% of 1-RM, with 100% of 1-RM being superior to the other loads when the loads were lifted at maximal intended velocity. However, the timing of maximal muscle activations was not affected by the different loadings for the quadriceps, but the timing was sequential and independent of loading (rectus femoris before vastus medial before vastus lateral). Maximal activation in the gluteus and semitendinosus increased with increasing loads. This means that for muscle activation, maximal lifting velocity may compensate for increased loads, which may allow resistance-trained athletes and individuals in rehabilitation to avoid heavy loads but still get the same muscle activation.

## Introduction

In resistance training, the free-weight back squat is frequently used to increase strength of the lower body. Different percentages of one-repetition maximum (% of 1-RM) are used to improve different muscle properties such as increasing maximal strength, explosive strength, and hypertrophy [1–3]. Different loads (% of 1-RM) result in different neuromuscular adaptations and lifting kinematics [4, 5]. Heavy loads (> 80% of 1-RM) have been used to recruit high-threshold fast-twitch motor units according to the size principle [5, 6], whereas lighter loads (30%–60% of 1-RM) have been used to maintain training speed specificity and enhance mechanical power output [5, 7]. However, performing ballistic movements with lighter loads could lead to a lower recruitment threshold and therefore recruit the high-threshold motor units [8]. Furthermore, previous studies have demonstrated that peak and average velocity decrease with increasing external load [9, 10]. The joint and barbell kinematics change with increasing numbers of repetitions, and the occurrence of peak velocity changes when fatigued [11, 12]. However, there have been limited studies examining neuromuscular activity and kinematics when the participants were asked to accelerate different loads (% of 1-RM) at maximum intended velocity.

Increasing the external load increases the demands of the muscles to produce enough force to complete the lifts and also increases the chances of accidents [5, 13]. A study that investigated muscle activation during lifts until full exhaustion [12] found that activation increased most from the first to the second and third repetitions in 6-RM squats and was kept stable in the last three repetitions. However, because that study involved only five muscles (vastus lateralis and medialis, rectus femoris, biceps femoris, and erector spinae), it does not offer full insights about the behavior of muscles during squats. Furthermore, the studies involving muscle activation during squats were all performed with a load above 80% of 1-RM, with several repetitions of lifts and with no directive to lift with maximal intended velocity [12, 14–17]. Two studies have investigated muscle activation during squats with loads varying from 60%, 75%, and 90% of 1-RM [18, 19]. However, in these two studies, different variations of back squats were compared with each other: with and without knee wraps [18], or the overhead with the standard squat [19], and not the different loads with each other.

In heavy resistance training (> 80% of 1-RM), kinematics and muscle activation have been examined. The majority of the previous studies have included explosive parameters (i.e., jump height, power output, rate of force development) but not kinematics and in-depth analyses of muscle activation in training regimes over the whole spectrum of loads including lower loads (30%–60% of 1-RM). Therefore, little is known about muscle activation and timing of maximal muscle activation comparing different loads (30%–100% of 1-RM) with maximum lifting velocity. The aim of this study, then, is to compare muscle activation patterning and barbell kinematics in free-weight back squat with different loads in experienced resistance-trained athletes. We hypothesize that muscle activity of the measured muscles will increase only after 60% of 1-RM (size-principle) and that upward phase duration will increase together with decreased maximal velocity.

## Materials and methods

### Participants

Thirteen healthy males experienced with resistance training were recruited from the local fitness center at the university college (aged 24.2 ± 2.0 years, body mass 81.5 ± 9.1 kg, height 1.78 ± 0.06 m, experience 6.3 ± 3.2 years). Inclusion criteria were being able to lift 1.5 times their own body weight (133.8 ± 16.7kg) in 1-RM squat (femur parallel to the floor) and no injuries or pain that could reduce their maximal performance. None of the participants were competitive powerlifters or weightlifters. The participants did not conduct any resistance training of the legs 72 hours before testing. Each participant was informed of the testing procedures and possible risks, and written consent was obtained prior to the study. The study complied with the current ethical regulations for research and approved by the Regional Committee for Medical Health and Research Ethics in Norway (REK Sør-Øst) and the Norwegian Centre for Research Data, in conformance with the latest revision of the Declaration of Helsinki.

### Procedures

The participants started with a standardized, progressive, specific warm-up protocol according to Saeterbakken and Fimland [20]. After a general warm-up on a treadmill or cycle, the protocol consisted of 15 repetitions at 30%, 10 repetitions at 50%, and 6 repetitions at 80% of the participants’ self-reported 6-RM loads in squatting. After the warm-up, free-weight back squats were performed. The free-weight back squat was performed in a power rack (Gym 2000, Modum, Norway) with an Olympic barbell (diameter = 2.8 cm, length = 1.92 m). The exercise started with fully extended knees and a natural sway in the lower back, which was maintained throughout the entire execution. Using a self-paced but controlled tempo, the participants lowered themselves to 80° knee flexion (180° fully extended knee) measured with a protractor (femur–fibula). When the participants had the correct knee angle, a horizontal elastic band was adjusted [20, 21]. The participants had to touch the band (mid-thigh) in every repetition before starting the concentric phase. A test leader gave oral confirmation when the participants touched the band. Before starting the tests using the different loads, 1-RM in free-weight back squat was performed. After the final warm-up set, the load was increased to approximately 95% of the participants’ self-reported 1-RM. The load was then increased by 2.5–5.0 kg until failure. Failure was defined by the following criteria: 1) the participants failed to complete a lift, 2) the participants could not complete the lift with proper technique, or 3) both the participant and the test leader agreed that the participant would not be able to lift 2.5 kg more. The 1-RM was achieved within 2–4 attempts. Each attempt was separated by a pause of 4–5 minutes. After the final 1-RM attempt, a 10-minute pause was given before starting the testing using the different loads. The loads began from 30%, with 10% increments until 100% of 1-RM, which was based on 1-RM achieved by each participant. Importantly, the participants were instructed to accelerate the loads in the entire concentric movement, which resulted in a jump using the lowest loads (i.e. 30%-60% of 1-RM). Two experienced test leaders ensured that the participants did not land with the barbell on their neck. The different loads were randomized for each participant, with random order determined by a random number generator. Two repetitions per load from 30% to 60% were conducted, while from 70% to 100%, 1 repetition per load was performed. A rest of 3–5 minutes was given between each attempt [22].

### Measurements

Wireless electromyography (EMG) was recorded by using a Musclelab 6000 system and analyzed by Musclelab v10.5.67 software (Ergotest Technology AS, Langesund, Norway). Before placing the gel-coated self-adhesive electrodes (Dri-Stick Silver circular sEMG Electrodes AE-131, NeuroDyne Medical, USA), the skin was shaved, abraded, and washed with alcohol. The electrodes (11 mm contact diameter and 2 cm center-to-center distance) were placed along the presumed direction of the underlying muscle fiber according to the recommendations by SENIAM [23, 24]. The electrodes were placed on the right leg on the muscle belly of the biceps femoris, semitendinosus, gluteus maximus, rectus femoris, and lateral and medial vastus. To minimize noise from the surroundings, the raw EMG signal was amplified and filtered using a preamplifier located close to the sampling point. The EMG signals were converted to root mean square (RMS) EMG signals using a hardware circuit network (frequency response 20–500 kHz, averaging constant 100 ms, total error ± 0.5%). The mean and peak RMS EMG signals of each muscle during the upward phase of the lift with each load were used for further analysis. The beginning and end of each lift were identified by using a linear encoder (ET-Enc-02, Ergotest Technology AS, Langesund, Norway) attached at the inside of the weights to the barbell. The encoder measures the upward phase duration of the barbell with a resolution of 0.075 mm and counts the pulses with 10-ms intervals [25]. Peak and average velocity of the barbell and time to peak velocity during the upward phase was calculated by using a 5-point differential filter with Musclelab v10.73 software (Ergotest Technology AS, Langesund, Norway).

### Statistical analysis

To assess the differences in EMG activity during the upward phase of the different loaded squats, a one-way analysis of variance (ANOVA) 1 x 8 (percentage of 1-RM: 30–100) with repeated measures was used. If significant differences were found, a Holm–Bonferroni post-hoc test was performed. In cases where the sphericity assumption was violated, the Greenhouse–Geisser adjustments of the *p*-values were reported. To assess differences in timing of the barbell during the free-weight back squats testing with different loads, a one-way ANOVA with repeated measures (percentage of 1-RM) was used. A two-way ANOVA 6 (muscles) by 8 (percentage of 1-RM) with repeated measures was used to evaluate the timing of maximal muscle activation during the lifts. The level of significance was set at *p* ≤ 0.05. When *p* was between 0.05 and 0.10 it was indicated with a trend [26]. Statistical analysis was performed with SPSS version 23.0 (SPSS Inc, Chicago, IL). Effect size was evaluated with η^2^_p_ (Eta partial squared) where 0.01 < η^2^ < 0.06 constitutes a small effect, a medium effect when 0.06 < η^2^ < 0.14, and a large effect when η^2^ > 0.14 [27].

## Results

**Fig 1:**
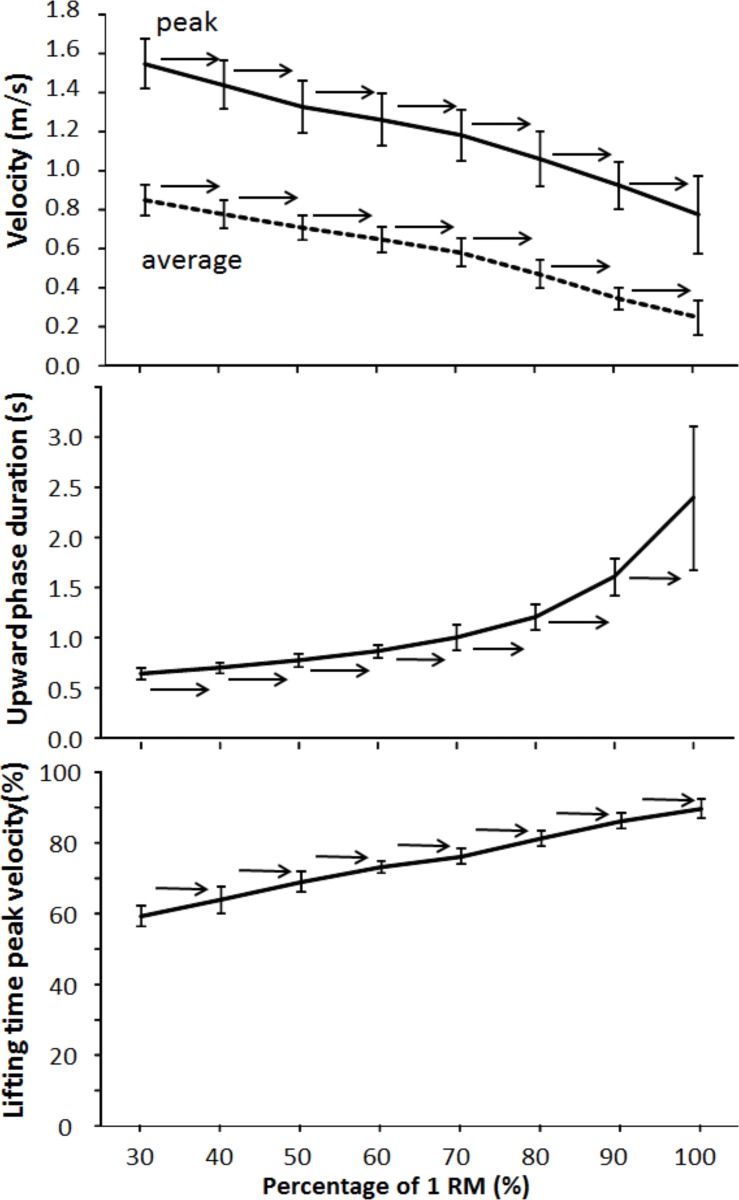
Mean (SD) average and peak velocity, upward phase duration, and relative time of occurrence of peak velocity in the upward phase at different percentages of 1-RM (30%–100%) of free-weight back squats. → indicates a significant difference (*p* ≤ 0.05) between this percentage and all percentages away from the sign.

The average barbell lowering velocity was approximately the same with all loads 1.7±0.4 s, except when with 1-RM load, which was significantly longer (1.98±0.45 s). The average and peak velocities changed significantly over lifted loads (F ≥ 75.8, *p ≤* 0.001; η^2^ ≥ 0.84). The post-hoc comparison showed that average and peak upwards lifting velocity decreased with each increase in lifting load (Fig 1). A significant change in upward phase duration (F = 59.5, *p <* 0.001, η^2^ = 0.84) was found with increasing lifting load (Fig 1). The post-hoc comparison showed that the upward phase duration significantly increased with each increasing load (Fig 1). Timing of peak velocity occurred later with each increasing percentage of 1-RM (Fig 1).

**Fig 2:**
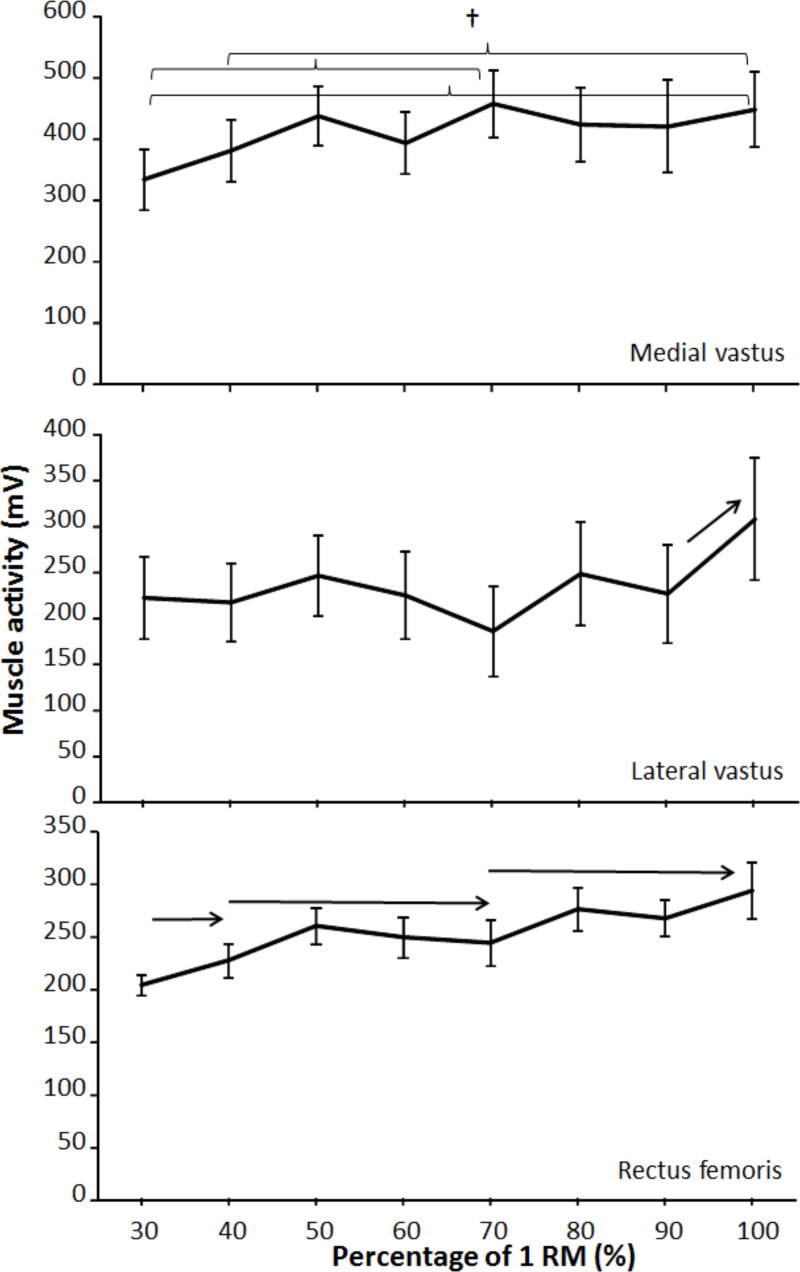
Mean (SD) root mean square (RMS) EMG activity for each percentage of the upward phase in vastus lateralis, vastus medialis, and rectus femoris during free-weight back squats. → indicates a significant difference (*p* ≤ 0.05) between this percentage and all percentages away from the sign. † indicates a significant difference (*p* ≤ 0.05) between these two percentages.

**Fig 3:**
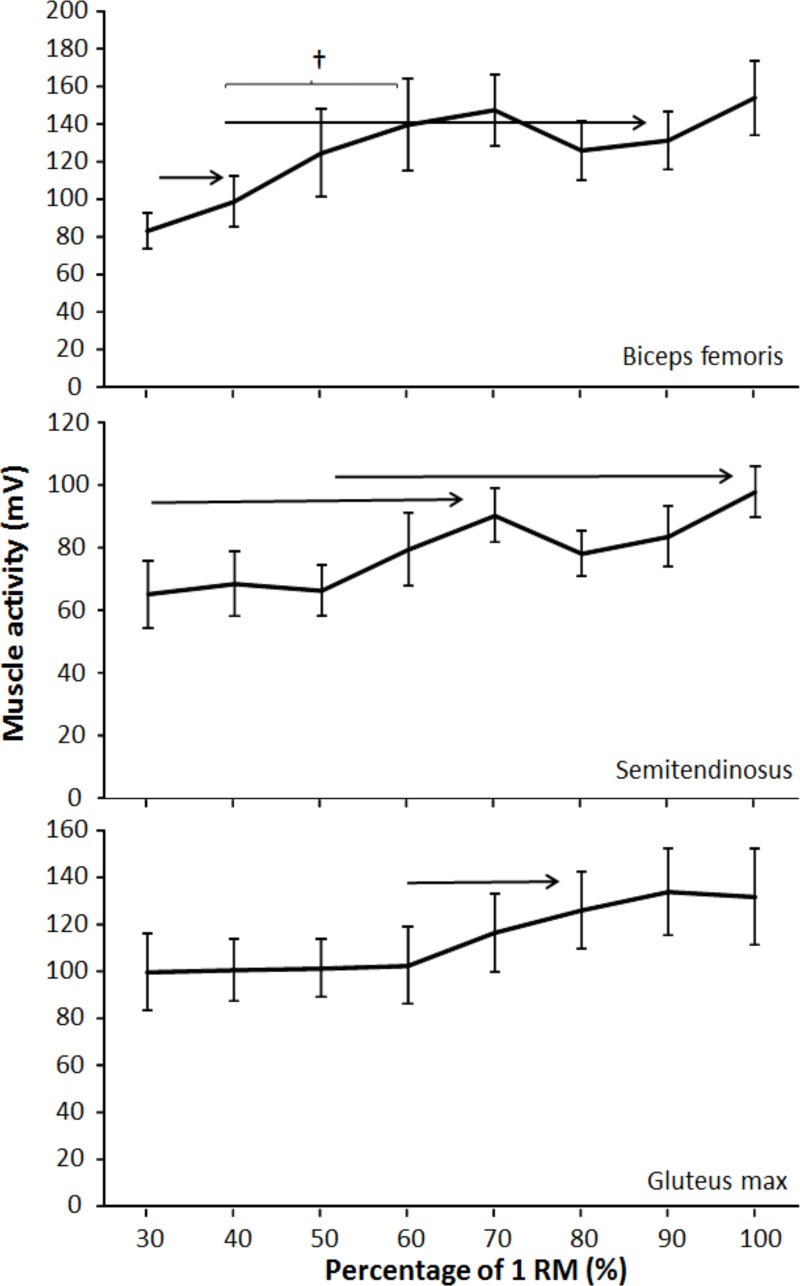
Mean (SD) root mean square (RMS) EMG activity for each percentage of 1-RM during upward phase in biceps femoris, semimembranosus, and gluteus maximus during free-weight back squats. → indicates a significant difference (*p* ≤ 0.05) between this percentage and all percentages away from the sign. † indicates a significant difference (*p* ≤ 0.05) between these two percentages.

A significant effect of lifting load was found for EMG activity for semitendinosus (F = 3.2 *p* = 0.049; η^2^ = 0.23) and rectus femoris (F = 5.0 *p* = 0.007; η^2^ = 0.31), while for the other four muscles, a trend (F ≥ 2.47, 0.054 < p < 0.08, η^2^ ≥ 0.18) was found. The post-hoc comparison indicated that with regard to the EMG activity, only the rectus femoris showed regular increases in activation with increasing load from 30% to 40%, 40%–70%, and 70%–100% of 1-RM (Fig 2). The medial and lateral vastus increased in activation only when performing 1-RM compared with the other loads (Fig 2), while gluteus maximus activity increased only between loads of 60%–80% of 1-RM (Fig 3). The semitendinosus increased activity between 30%–70% and 50%–100% of 1-RM loads (Fig 3), while biceps femoris increased in muscle activation between 30%–40% and 40%–90% of 1-RM loads (Fig 3).

**Fig 4:**
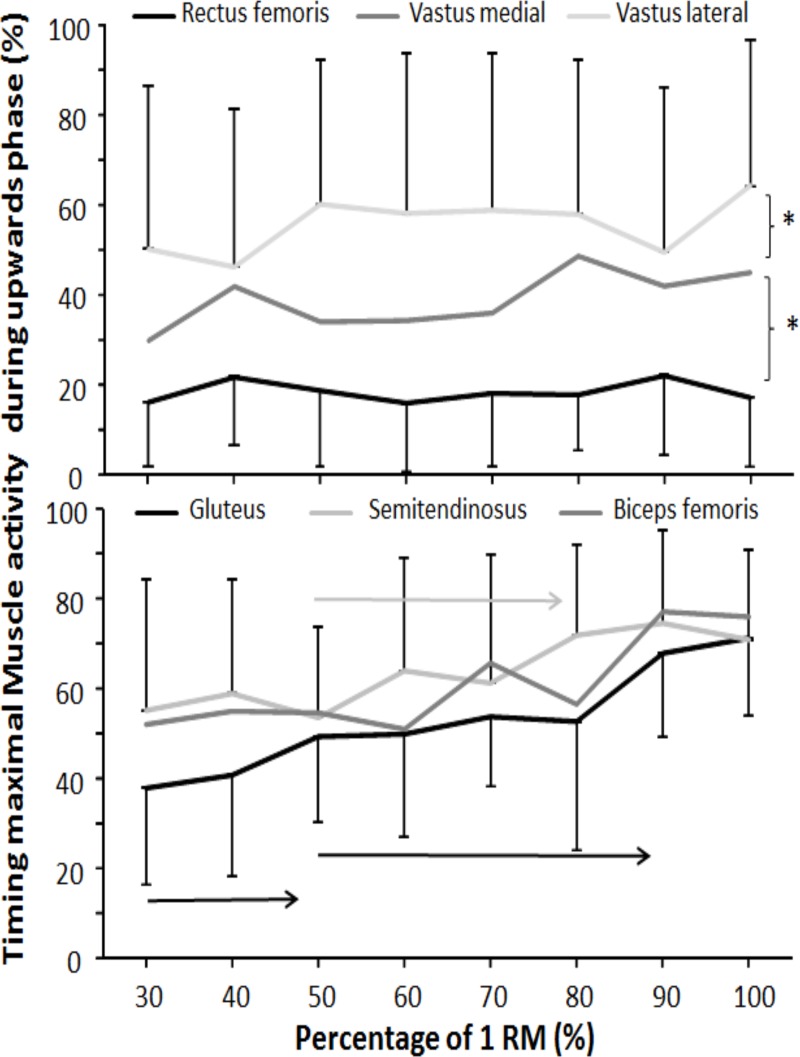
Relative time of occurrence of maximal RMS muscle activation at different percentages of 1-RM (30%–100%) of free-weight back squats. → indicates a significant difference (*p* ≤ 0.05) between this percentage and all percentages away from the sign for this muscle. * indicates a significant difference (*p* ≤ 0.05) between these two muscles in order of occurrence.

The time of occurrence of the maximal RMS of the different muscles showed that both percentage of 1-RM (F = 5.1 *p* < 0.005; η^2^ = 0.32) and muscles (F = 10.99 *p* < 0.001; η^2^ = 0.50) had an effect upon the occurrence of maximal RMS. Furthermore, a significant load*muscles interaction was found (F = 1.54 *p* = 0.029; η^2^ = 0.12). The post-hoc comparison revealed that the occurrence of maximal muscle activation started with the rectus femoris (20% in upwards phase), followed by the vastus medial (40%). From the medial vastus, all other muscles appeared around 54% to 62%, with no significant difference in occurrence between these muscles (Fig 4). The post-hoc comparison of percentages revealed that the timing of semitendinosus changed only from loads with 50% to 80% of 1-RM, and for gluteus maximus, timing changed from 30% to 50% and again from 50% to 90% of 1-RM, which also causes to interaction effect.

## Discussion

The aim of this study was to compare barbell kinematics and muscle patterning in free-weight back squatting with different loads, but with maximum lifting velocity, in young males with resistance training experience. Average and peak upwards lifting velocity decreased, while upward phase duration increased, with each increasing load (Fig 1). Timing of peak velocity occurred later with each increasing percentage of 1-RM. The timing of maximal muscle activations was not affected by the different loadings for the quadriceps, but the timing was sequential and independent of loading (rectus femoris before vastus medial before vastus lateral). The maximal activation in the gluteus maximus and semitendinosus increased with increasing loads. In general, the muscle activation in all muscles increased with increasing loads but was not linear.

With increasing loads, the barbell velocity decreased, the upward phase duration increased, and the peak velocity occurred later. The results were as hypothesized and supported by previous studies [9, 10]. The force–velocity relationship demonstrated in the present study is not surprising and could be explained by the laws of Newton (F = m x a). The acceleration is then the force divided by the weights lifted. With increasing loads, but with approximately similar maximal force in each lift (the participants were instructed to lift at maximal intended velocity), the acceleration had to decrease with increasing loads. This could also explain the occurrence of peak velocity later in the movement with increasing loads. With the lowest loads (30% of 1-RM), the peak velocity was observed at 60% of the barbell displacement upwards, while the peak velocity with the highest loads (1-RM) was observed at 90%. With greater loads, the acceleration was lower in the beginning, resulting in lower velocity and longer upward phase duration, which eventually resulted in the peak velocity appearing later in the movement with increasing loads [28, 29]. Furthermore, with heavy load (> 85% of 1-RM) or fatigue, the sticking region occurred, which causes a longer upward phase duration [12, 15, 16]. In addition, shorter lever arms and more cross-bridges between contractile filaments later in the movements may also explain the kinematics in these findings [30–33].

The relationship between muscle activity and increasing loads has been shown to be close to linear in isometric contraction and in dynamic contraction [34, 35]. In the present study, increasing muscle activation was observed with increasing loads but was not linear. For example, there were no differences between loads 30%–90% of 1-RM in vastus lateral, 40%–60% and 70%–90% in vastus medial, and 50%–90% in rectus femoris. However, in the quadriceps, greater muscle activation was observed performing 1-RM compared to the other loads. For the gluteus maximus, greater muscle activation was observed for the loads 80%–100% of 1-RM, but only compared to the loads 30%–60% of 1-RM. These results corroborate those by Yavuz and Erdag [17] and Gomes et al. [18], who also found increases in gluteus maximus activity with increasing loads. The results in vastus lateral were in contrast to previous findings. Gomes et al. [18] reported an increase comparing 60%–90% of 1-RM loads, and Yavuz and Erdag [17] reported increases in the vastus medial between 80% and 90% of 1-RM loads. The discrepancy in findings in some of the muscles with these previous studies could be the result of experience (3 years vs. 6 years of resistance training experience) and strength level (107 and 120 kg as 1-RM compared with 130 kg in 1-RM in the present study). One could speculate that greater performance level in the present study could be related to longer training experience. This might suggest a better muscle recruitment and firing frequency strategy testing the spectrum of different loads [36, 37] and explain the inconsistent results compared to previous studies [17, 18].

To the authors’ knowledge, this is one of the few studies examining muscle activation with increasing loads in squats where the participants were instructed to lift at maximal intended velocity over a large spectrum of loads. In comparison, Cochrane and Barnes [38] examined muscle activation in deadlift with 30%, 40%, 50%, and 75% of 1-RM and also found no differences between the loads in the biceps femoris or gluteus maximus. In contrast to the present study, Pincivero et al. [35] examined muscle activation in the quadriceps in knee extension with increasing loads (20%–90% of 1-RM) and found a near perfect linear relationship between muscle activation and loading, which was also reported during isometric contraction in single-joint and multi-joint exercises in the quadriceps [34]. However, none of these studies examined the influence of movement velocity, which most likely can explain the contradictory findings from the present study. However, the present study corroborates results found by McBride et al. [4], who examined vastus lateral activation in squats using 70%, 80%, and 90% of 1-RM. The participants were instructed to lift at maximal intended velocity. Even though the aim of that study was not to compare the muscle activation between loads, the difference between 70% and 90% of 1-RM was only 1.3%.

For the antagonist biceps femoris and semitendinosus in the upward movement, there were no differences between the loads 60%–100% of 1-RM. Still, lifting 70%–100% of 1-RM demonstrated greater muscle activation than the lowest load (30% of 1-RM). The results were not surprising in terms of hamstring muscles being an antagonist in the upward movement and therefore to a lesser extent being affected by the loading. To the authors’ knowledge, no previous studies have examined the antagonistic activation in squats with increasing loads. Increased muscle activation using loads above 70% of 1-RM may be the result of co-contraction to stabilize the knee and pelvis in the turnover from eccentric to concentric movement. The hamstring muscles contribute to avoiding a forward rotation of the pelvis. While an increased activation of the rectus femoris would increase the hip flexor torque, the activation of the hamstring may be of more importance as the loads increase and lifting velocity decreases [39].

The present study found a sequential and significant difference in maximal peak activation between the quadriceps muscles starting with rectus femoris, vastus medial, and then vastus lateral. The peak activation pattern was independent of loads and fairly constant (see Fig 4). The peak activation occurred at approximately 85°–103° knee flexion, as shown by van den Tillaar [15]. The findings were partly supported by a previous study by Escamilla et al. [40]. They demonstrated a peak activation at approximately 100°–110° knee flexion for the quadriceps muscles, examining 12-RM loads among experienced participants. However, the 12-RM loads were lifted in a slow and continuous manner (1–1.5 seconds in the upward phase), which may explain the minor variation in peak activation. Yet Escamilla et al. [40] did not report any differences in maximal timing between the quadriceps muscles. The quadriceps muscles component may provide a different contribution to knee extensor torque due to their anatomical structure [30, 31]. For example, the rectus femoris has a bi-articular function as a hip flexor and knee extensor [39, 41]. The rectus femoris may therefore be the first muscle to activate to stabilize the hip. A later timing of peak activation may thereby increase the torque of the hip.

The gluteus maximus demonstrated differences in the timing of maximal activation between 30% and 50% of 1-RM and from 50% and 90% of 1-RM. The change in timing may be the result of lower lifting velocity with increasing loads. The participants were more dependent on the contributions and coordination between the different prime movers, in contrast to lighter loads where the participants had a rapid acceleration from the lowest position. To the best of our knowledge, no previous study has examined the timing of gluteus using different loads. However, several studies have examined the peak hamstring muscles (biceps femoris and semitendinosus) and reported the peak to be between 110° and 130° knee flexion [39, 40]. The findings of the present study support these previous studies. However, the biceps femoris demonstrated similar maximal timing across the loads, while the semitendinosus had significant later maximal timing between 50% and 80% of 1-RM. Greater coactivation with the heaviest loads (> 80% of 1-RM) may avoid a hip flexion torque caused by the rectus femoris activation with increasing loads [39, 42].

A limitation of the present study is that only resistance-trained males were included, and the results may therefore not be generalized to other populations. Furthermore, there is always a risk of cross talk from nearby muscles using surface EMG, which thereby would generate inaccurate measurements. Finally, the study did not include measurements of peak or angle velocity of the ankle, knee, or hip, and no analysis was performed on different parts of the upward phase, which could demonstrate different techniques testing with the different loads.

### Practical implications

The present study included resistance-trained males, and the results may therefore not be generalized to other populations. Based on the present study, resistance-trained athletes may decrease the loads but have similar muscle activity when lifting with maximal lifting velocity. By decreasing the loads, the mechanical stress decreases and time to recover is reduced. Using lower loads with maximal lifting velocity may therefore allow athletes to increase the total volume without increasing the risk of injuries. With the exception of the heaviest load (1-RM), the prime movers (quadriceps and gluteus maximus) have similar muscle activations between 70% and 90% of 1-RM and between 40% and 60% of 1-RM. Therefore, athletes and trainers could vary the loading within the load windows and expect the same effect. This is important in regard to accommodating athletes’ preferences. Furthermore, the force requirement differs during different tasks/sports, and a variation in the loading could help to deal with this differentiation.

## Conclusions

With increasing load, average and peak upwards lifting velocity decreased, while upward phase duration increased together with a later occurrence of peak velocity. In general, similar muscle activations in the prime movers were observed for loads between 40% and 60% of 1-RM and between 70% and 90% of 1-RM, with 100% of 1-RM being superior to the other loads when the loads were lifted at maximal intended velocity. This means that maximal lifting velocity may compensate for increased loads, which may allow resistance-trained athletes and those in rehabilitation (resistance-trained athletes) to avoid heavy loads but still get the same muscle activation.

## Supporting information

S1 File EMG and kinematic data. (XLSX)
